# Targeting Myd88 using peptide-loaded mesenchymal stem cell membrane-derived synthetic vesicles to treat systemic inflammation

**DOI:** 10.1186/s12951-022-01660-x

**Published:** 2022-10-15

**Authors:** Kyong-Su Park, Markus Bergqvist, Cecilia Lässer, Jan Lötvall

**Affiliations:** Krefting Research Centre, Institute of Medicine, Sahlgrenska Academy, University of Gothenburg, Gothenburg, Sweden

**Keywords:** Engineered extracellular vesicles, Synthetic eukaryotic vesicles, Mesenchymal stem cells, Systemic inflammation, Anti-inflammation, Drug delivery, Therapy

## Abstract

Mesenchymal stem cells (MSC) secrete extracellular vesicles (EV) with a regenerative profile, and an increasing number of studies have focused on the utilization of MSC-EV for therapeutic drug delivery. However, EV are usually produced by cells in low quantities and are packed with numerous cytoplasmic components, which may be unfavorable for further drug loading. In this study, we developed a simple process for generating membrane vesicles directly from the cells, which we refer to as synthetic eukaryotic vesicles (SyEV). We hypothesized that MSC-derived SyEV can be efficiently loaded with an anti-inflammatory drug and the loaded vesicles can strongly suppress the systemic inflammation induced by bacterial outer membrane vesicles (OMV). SyEV were generated from MSC membranes through serial extrusion of the cells, ionic stress, and subsequent vesiculation of the membrane sheets, leading to high yield and purity of the SyEV with few cytosolic components remaining. When these SyEV were given to macrophages or mice exposed to OMV, the release of pro-inflammatory cytokines was similarly attenuated comparable to treatment with natural EV. We then loaded the SyEV with large numbers of peptides targeting Myd88 and observed enhanced therapeutic potential of the loaded vesicles in OMV-induced macrophages. Further,* in vivo *experiments showed that the peptide-encapsulated MSC-SyEV suppressed cytokine production synergistically. Taken together, these findings suggest that SyEV-based therapeutics is a highly interesting platform for delivering an advanced therapeutic drug for the treatment of systemic inflammation without severe side effects.

**Supplementary Information:**

The online version contains supplementary material available at 10.1186/s12951-022-01660-x.

## Introduction

Mesenchymal stem cells (MSC) are multipotent cells that can be isolated from multiple human tissues such as bone marrow, adipose tissue, or umbilical cord blood. Due to their highly regenerative ability, there are increasing studies that have reported their immunomodulatory potential in various inflammatory diseases, including sepsis [1, 2]. Also, MSC have been engineered to deliver therapeutic drugs because these cells are able to home in to injured tissue [3]. However, the use of whole cells as a drug delivery vehicle still faces many limitations – including the potential of malignant transformation of the live cells as well as their low production and strict storage conditions – that ultimately limit their clinical applicability. Recently, pre-clinical studies have suggested that extracellular vesicles (EV) secreted from MSC carry over the therapeutic activity of the originating cells, which may overcome many of the critical issues of using MSC for drug delivery [4, 5].

EV are naturally released by all eukaryotic cells, including MSC, and are morphologically spherical proteolipid structures with a diameter of 30–3000 nm [6, 7]. The vesicles contain biologically active proteins, lipids, and genetic material that can be delivered to a recipient cell in a functional form [8]. Importantly, the transfer of functional molecules can influence the recipient cell phenotype through multiple mechanisms [9]. EV may be utilized as therapeutics, especially by exploiting their ability to deliver bioactive molecules to the inside of the cell, which is especially useful for delivering molecules that do not readily pass across cell membranes. This concept has inspired researchers to develop technologies to load EV with different therapeutic molecules, including siRNA, mRNA, and proteins, using technologies such as electroporation and intracellular cell engineering [10, 11]. However, the loading capacity of the EV platform is modest, and EV are generally produced in relatively low quantities by cells, leading to a critical limitation in scaling up the production of vesicles for clinical use [12].

In order to reduce the hurdles to applying EV in the clinic, we recently developed EV-mimetic nanovesicles (NV) that can be artificially generated from MSC membranes through a serial extrusion process that leads to a much higher yield of vesicle production [13]. These extruded NV have a significant therapeutic inhibitory effect on systemic inflammation and lung inflammation in mice. However, the extruded NV may not be an optimal drug vehicle platform because they carry unnecessary cytoplasmic molecules, including nuclear proteins and nucleic acids such as RNA and DNA. Given that ionic stress can open membranes [14, 15], we exploited the procedure to remove cytoplasmic molecules from the NV, resulting in a therapeutic vesicle that has fewer unwanted molecules and therefore a greater loading potential. After this procedure, the empty membrane vesicles are referred to as synthetic eukaryotic vesicles (SyEV).

In this study, we hypothesized that SyEV can be directly generated from MSC membranes through a series of processes that includes cell extrusion, ionic stress, and mild sonication for vesicle manufacturing and that these engineered SyEV can be deployed as an intrinsically bioactive drug vehicle platform to attenuate inflammation. To test this hypothesis, we developed MSC-derived SyEV loaded with a specific anti-inflammatory peptide targeting Myd88, a common intracellular signaling molecule downstream of most Toll-like receptors (TLRs). To test the enhanced protective role of these SyEV, we induced a systemic inflammation by administration of bacterial outer membrane vesicles (OMV), which are known to induce a sepsis-like systemic inflammation and multiple organ dysfunction [16, 17].

## Results

### MSC-derived SyEV have increased purity and reduced cytosolic components compared to extruded NV

**Fig. 1:**
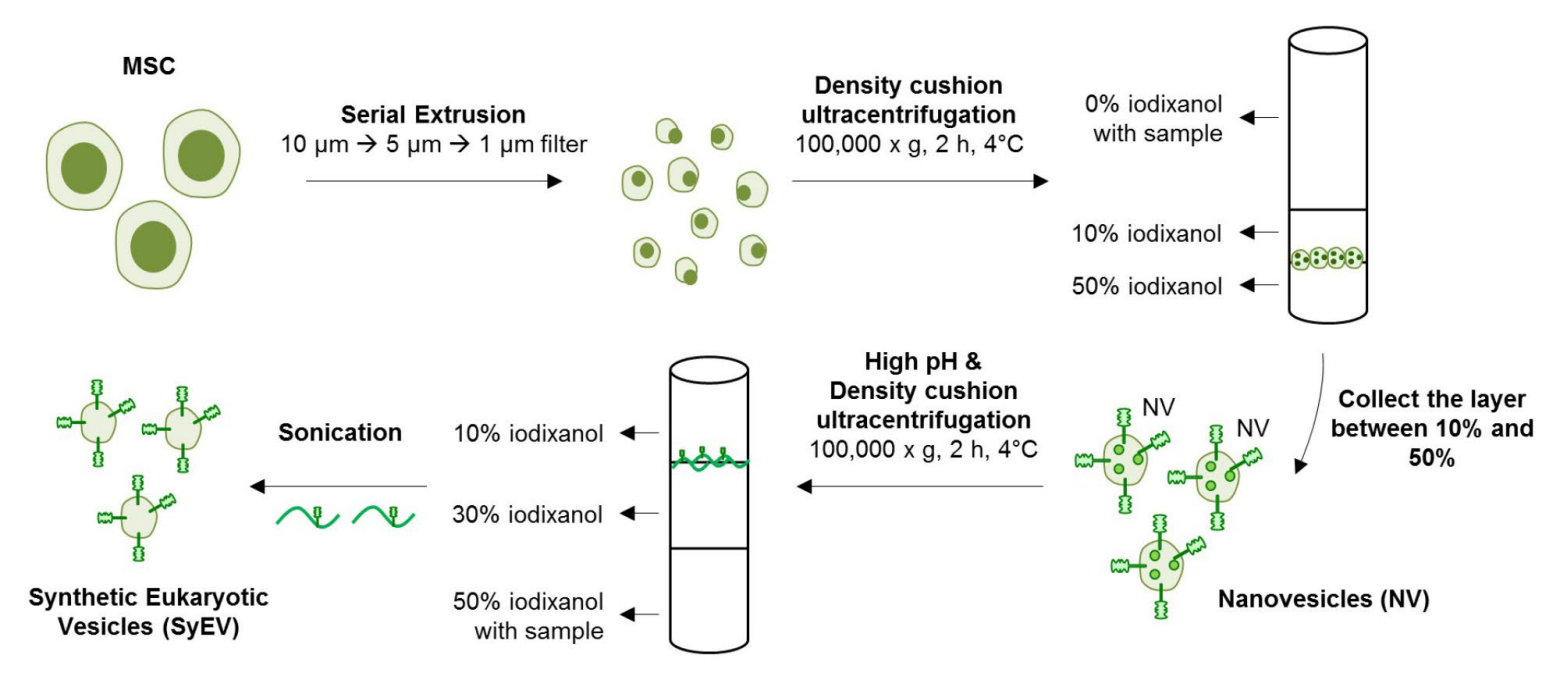
Schematic diagram of the generation of SyEV directly from MSC-derived membranes. MSC membranes were isolated by serial extrusion and ionic stress, and pure SyEV were then generated by mild sonication

**Fig. 2:**
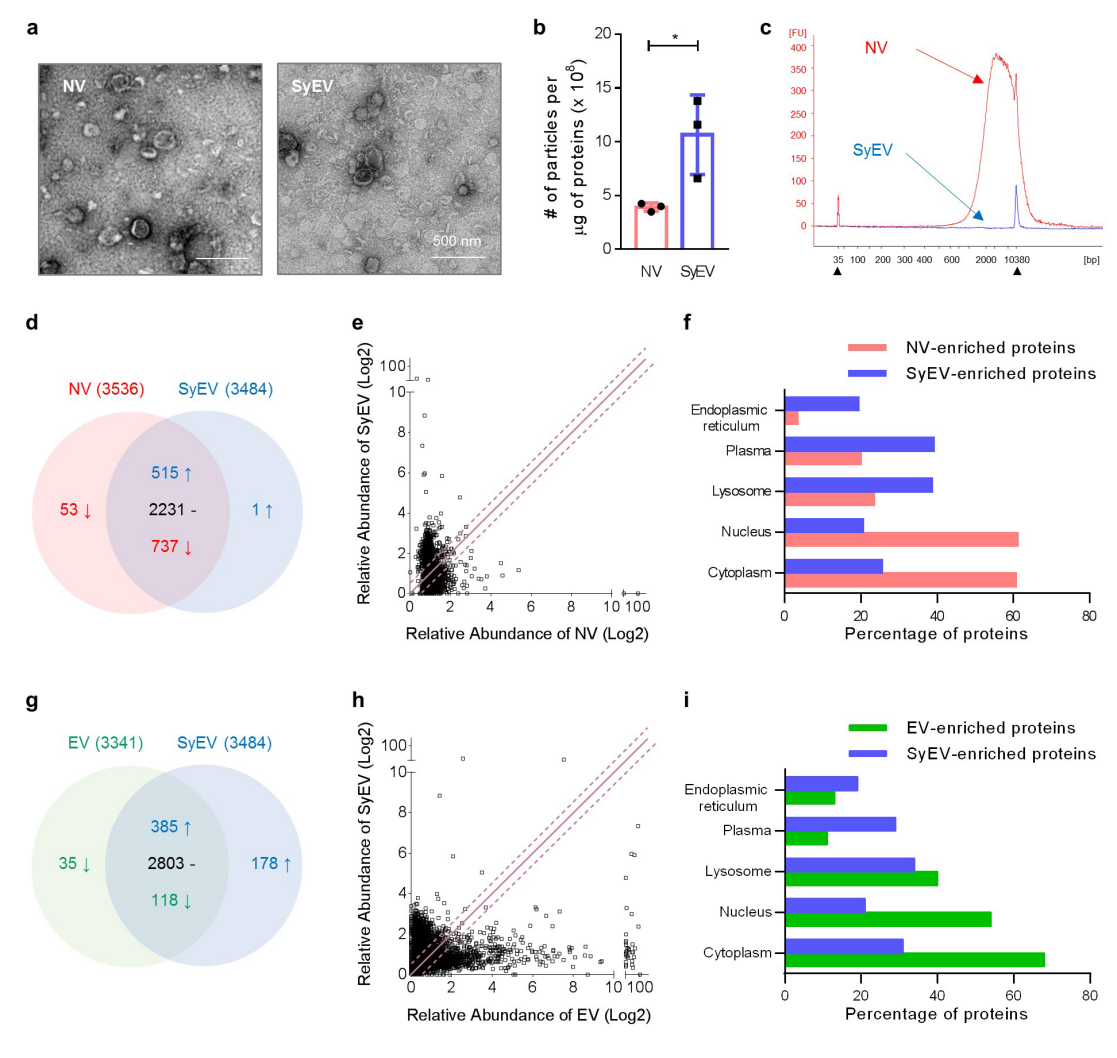
Characterization of MSC-derived SyEV. **a** TEM image of the generated NV and SyEV. Scale bars, 500 nm. **b** The number of particles per one microgram of vesicular proteins (*n* = 3). Data are presented as the mean ± SEM. *P < 0.05 by unpaired two-tailed Student’s *t*-test. **c** Representative electropherograms of DNA molecules derived from SyEV in comparison to those from NV. Filled triangles indicate internal markers. **d** Venn diagram of MSC-derived NV and SyEV proteomes. The common proteins are divided into three groups (1.5-fold increase, 1.5-fold decrease, and no change) based on the relative protein abundance (*n* = 2). **e** Plot of the log2 value of the relative protein abundance from NV and SyEV. The solid line and dotted lines show no change and 1.5-fold change, respectively. **f** Different GO cellular components were compared between the NV and SyEV proteome groups. **g** Venn diagram of MSC-derived EV and SyEV proteomes. The common proteins are divided into three groups (1.5-fold increase, 1.5-fold decrease, and no change) based on the relative protein abundance (*n* = 2). **h** Plot of the log2 value of the relative protein abundance from EV and SyEV. The solid line and dotted lines show no change and 1.5-fold change, respectively. **i** Different GO cellular components were compared between the EV and SyEV proteome groups

SyEV were generated directly from MSC-derived membranes according to the procedure described in the Methods section and shown in Fig. 1. The manufacturing process was modified from our previously published method [13]. Briefly, extruded NV were first produced by forcing cells through a series of small pore-sized polycarbonate membrane filters (10 μm, 5 μm, and 1 μm). The isolated NV were exposed to an alkaline solution (pH 11) to open the vesicle structure, and then the membrane sheets were collected from the interface layers between the 10% and 30% iodixanol layers following iodixanol-based density cushion ultracentrifugation. Finally, the purified membranes were converted into SyEV by mild sonication (Fig. 1). The SyEV were visualized by transmission electron microscopy (TEM) and showed EV-like closed spherical structures (Fig. 2a), and nanoparticle tracking analysis showed nanosized particles with an average diameter of 125.6 ± 5.7 nm (Additional file 1: Fig. S1), and these were similar to the morphological characteristics of NV. Nanoparticle tracking analysis also showed that the SyEV were three times purer than NV with regards to the particle number per µg of vesicular protein (Fig. 2b). Also, DNA and RNA molecules included in the SyEV were almost totally removed during the vesicle manufacturing process, whereas NV still harbored large quantities of DNA and RNA (Fig. 2c and Additional file 1: Fig. S2).

We next examined the differences in protein composition of SyEV compared to NV. Quantitative proteomic analysis identified 3536 and 3484 vesicular proteins from NV and SyEV, respectively. As shown in the Venn diagram (Fig. 2d), 3483 proteins were identified in both vesicle preparations, whereas 53 and 1 protein were uniquely identified in NV and SyEV, respectively. The analysis of the relative abundance of different proteins indicated that 2231 proteins did not change markedly in abundance among the 3483 proteins, however 515 and 737 proteins were relatively increased (1.5-fold) or decreased (1.5-fold) in SyEV compared to NV (Fig. 2d, e). The subcellular localization of the enriched NV and SyEV proteins was determined by GO term analysis (Fig. 2f), and the SyEV had relatively fewer cytosolic and nuclear proteins and were instead mainly composed of a high portion of plasma membrane, endoplasmic reticulum, and lysosomal proteins, thus indicating the enrichment of membrane proteins in the SyEV.

We have previously shown that NV carry specific EV marker proteins such as tetraspanins (CD9, CD63, and CD81) and Flotillin-1 [13]. Interestingly, nanoparticle flow cytometric analysis of SyEV showed the presence of CD63 on the vesicular surface (Additional file 1: Fig. S3), and other EV tetraspanins were also found in the SyEV proteome (data not shown), showing that SyEV share a common protein profile with EV. Moreover, we performed comparative proteomics to determine in greater detail the protein composition of SyEV vs. EV. The relative abundance of each protein showed that 2803 proteins were not altered between SyEV and EV, whereas 563 and 153 proteins were increased and decreased in SyEV, respectively (Fig. 2 g, h). Further, the SyEV proteome was relatively enriched in subcellular localization proteins belonging to the plasma membrane, while cytosolic and nuclear proteins were less abundant compared to EV (Fig. 2i), which was similar to the comparison of SyEV vs. NV (Fig. 2f). Overall, these findings suggest that our specific procedure for SyEV produces highly pure membrane vesicles deficient in cytosolic proteins but enriched in membrane proteins.

### Functional proteomic profiling of SyEV predicts their ability to modulate the immune system

We additionally performed GO analysis using the Panther tools to understand the therapeutic function driven by SyEV proteins. Proteins of the SyEV proteome were primarily transporters, transmembrane signal receptors, and cell adhesion molecules (Additional file 1: Fig. S4a). Importantly, several subsets of proteins were predicted to be involved in cellular defense/immunity, supporting the hypothesis that SyEV might retain anti-inflammatory functions. Further functional analysis of the SyEV proteome revealed that many proteins belong to biological pathways regulating inflammation and cytokine/chemokine biology (Additional file 1: Fig. S4b). Moreover, the Funrich analysis tool showed that SyEV proteins were enriched in the GO biological processes of signal transduction, cell communication, and transport, whereas SyEV proteins were less related to cell growth and metabolism (Additional file 1: Fig. S4c).

### MSC-derived SyEV retain the intrinsic anti-inflammatory function of NV or natural EV produced by the same cells

**Fig. 3:**
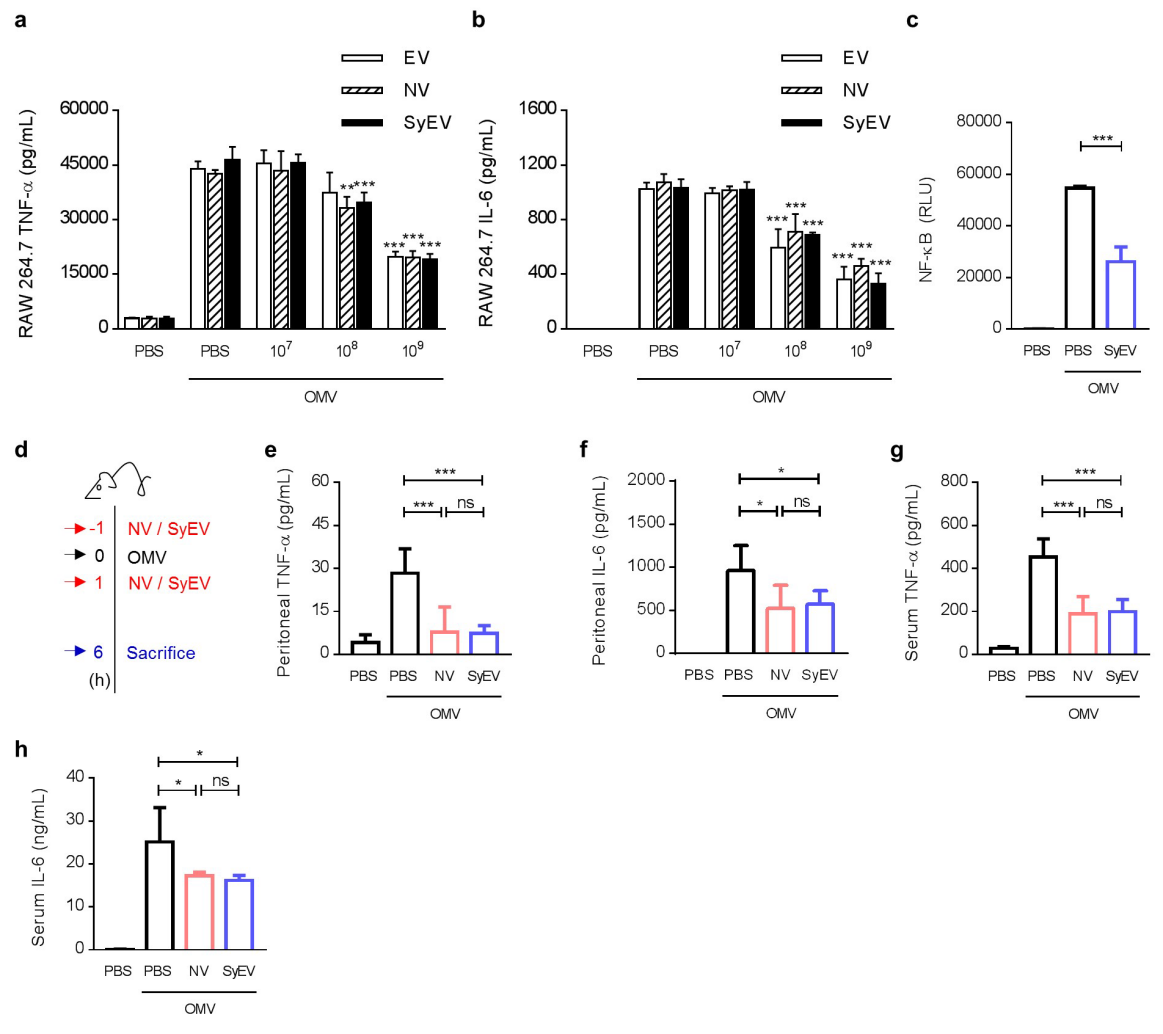
SyEV showed anti-inflammatory properties in OMV-treated macrophages and septic mice. **a, b** The inhibitory dose responses of EV, NV, and SyEV in OMV-activated RAW 264.7 cells. RAW 264.7 cells were pre-treated with OMV (100 ng/mL) for 3 h and then incubated with the same numbers of EV, NV, or SyEV particles for 15 h. The concentration of TNF-α (**a**) and IL-6 (**b**) was measured in the conditioned media (*n* = 3). **c** THP1-Lucia cells were treated with OMV (100 ng/mL) for 3 h to stimulate NF-ĸB signaling associated with luciferase, and the enzyme activity was measured as RLU (relative light units) following treatment with SyEV (1 × 10^9^) for 18 h (*n* = 3). **d** Experimental design for investigating the beneficial effect of NV and SyEV in vivo (*n* = 5). Mice were i.p. injected with NV or SyEV (2 × 10^9^) at 1 h before administration of a sublethal dose of OMV, and then the mice were again i.p. injected with the same dose of NV or SyEV at 1 h. Six hours after OMV injection, mice were sacrificed to measure inflammatory parameters. **e, f** The concentration of TNF-α (**e**) and IL-6 (**f**) in the peritoneal fluid. **g, h** The serum level of TNF-α (**g**) and IL-6 (**h**). Throughout, the data are presented as the mean ± SEM. ^*^P < 0.05, ^**^P < 0.01, ^***^P < 0.001; ns, not significant, by one-way ANOVA with Tukey’s post test (**c**-**h**) or two-way ANOVA with Tukey’s post test versus the OMV-only group (**a**-**b**)

Naturally produced EV from MSC have been shown to have a beneficial effect against sepsis [18], and MSC-derived NV can exert a significant anti-inflammatory effect on immune cells [13], thus we compared the therapeutic activity of SyEV with EV and NV regarding the anti-inflammatory effect on activated macrophages. We used the RAW 264.7 macrophage cell line as a model of inflammation and treated the cells with bacterial OMV to induce the release of pro-inflammatory cytokines such as tumor necrosis factor (TNF)-α and interleukin (IL)-6. Both SyEV, NV, and EV treatment blocked the release of pro-inflammatory cytokines from OMV-stimulated macrophages in a dose-dependent manner (Fig. 3a, b). The therapeutic potential of MSC-derived EV has been shown to be generally associated with various vesicular protein effectors [19], thus suggesting that there is a specific subset of functional vesicular proteins shared by SyEV, NV, and EV. This is interesting because SyEV have similar bioactivity as NV and EV despite significantly reduced cytosolic components inside the vesicles. To further determine the related signaling molecules, we determined whether SyEV could prevent the activation of nuclear factor-ĸB (NF-ĸB), which is a major signaling pathway for the production of pro-inflammatory cytokines [20], and inhibition of OMV-induced NF-ĸB stimulation was observed (Fig. 3c). Also, SyEV could significantly block the increase of IL-1β from OMV-stimulated macrophages (Additional file 1: Fig. S5a). Moreover, SyEV could increase more IL-10 secretion from the activated macrophages (Additional file 1: Fig. S5b), suggesting that IL-10 is one of the mediators for SyEV-induced anti-inflammation.

We next explored whether MSC-derived SyEV could also exert therapeutic effects in mice experiencing a systemic inflammation. Following a previously established severe inflammation model in vivo [16], a sublethal dose of OMV was intraperitoneally (i.p.) injected into mice pretreated with SyEV or NV for 1 h before OMV stimulation (Fig. 3d). After the OMV dose, the mice were i.p. administered once again with SyEV or NV for 1 h followed by sacrifice at 6 h to measure various inflammatory parameters. OMV-treated mice exhibited a decrease in body temperature and body weight, and this was significantly reduced by SyEV similar to the NV-treated group (Additional file 1: Fig. S6). Moreover, OMV-induced increases in TNF-α and IL-6 in the peritoneal fluid were significantly suppressed in both SyEV-treated and NV-treated mice (Fig. 3e, f), implying that SyEV retain anti-inflammatory therapeutic activity.

To further demonstrate the functional role of SyEV in systemic inflammation, we investigated the anti-inflammatory activity of the vesicles in the blood and lungs from OMV-treated mice. The administration of SyEV or NV efficiently reduced the elevation of TNF-α and IL-6 levels in blood observed after OMV exposure (Fig. 3 g, h), which was consistent with the cytokine profile in peritoneal fluid. During the progression of the systemic inflammation in sepsis, the overexpressed cytokines can cause multiple organ dysfunction, including the lungs, leading to multi-organ failure and death [21]. We therefore looked for changes in the numbers of leukocytes and cytokines in bronchoalveolar lavage (BAL) fluid as indicators of the inflammatory response in the lungs. Similar to the results confirmed in the blood, both SyEV and NV dampened the OMV-induced increase in the level of total infiltrated immune cells and cytokines (Additional file 1: Fig. S7), suggesting that SyEV maintain the strong inhibitory potential of NV on systemic inflammation even though cytosolic components are mostly eliminated by the SyEV manufacturing process. Also, we compared the influence of post-treatment of SyEV with pre-treatment. As a result, post-treatment with SyEV has also shown significant anti-inflammatory activity, however the inhibitory potential was not comparable to pre-treatment (Additional file 1: Fig. S8). Thus, the pre-treatment strategy was used in the following in vivo experiments.

### SyEV are efficiently internalized into macrophages via active endocytosis

**Fig. 4:**
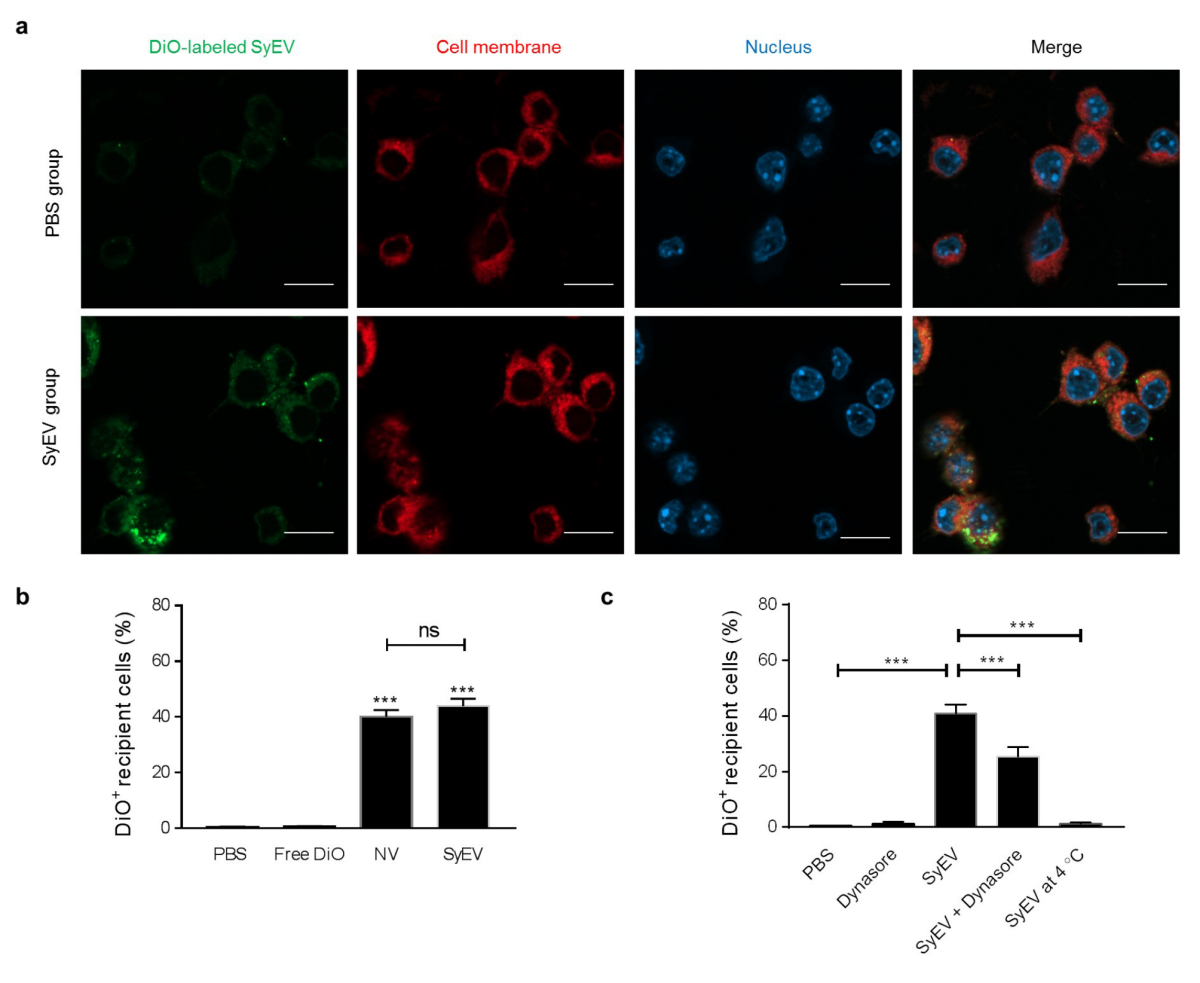
SyEV are taken up by macrophages through active endocytosis. **a** DiO-labelled SyEV (green; 1 × 10^9^) were incubated with RAW 264.7 cells for 6 h. Cell membranes and nuclei were stained by Cellmask Deep Red (red) and DAPI (blue), respectively. Scale bars, 20 μm. **b** The uptake of SyEV by cells was compared with NV by flow cytometry, and the results are shown as the percentage of DiO-positive cells (*n* = 3). **c** The macrophages were preincubated with Dynasore for 1 h at 37 °C, followed by treatment with DiO-labelled SyEV for 6 h at 37 °C. In parallel, SyEV were added to the cells for 6 h at 4 °C. The uptake of the labelled SyEV by cells was analyzed by flow cytometry (*n* = 3). Data are presented as the mean ± SEM. ^***^P < 0.001; ns, not significant, by one-way ANOVA with Tukey’s post test

We previously observed increased NV uptake by macrophages [13], so we next determined whether there was a difference in the uptake of SyEV and NV by the same cells. RAW 264.7 cells were treated with DiO-labeled SyEV for 6 h, and cellular uptake was monitored with confocal microscopy using the green fluorescence signal of SyEV showing that the vesicles were detected on the recipient cell membranes as well as inside of the cells (Fig. 4a). Incubation with free DiO dye did not lead to positive signals in cells, and there was no apparent difference in the cellular uptake of SyEV and NV according to the quantitative FACS analysis (Fig. 4b).

To elucidate the underlying mechanism of the uptake of SyEV, we pre-treated cells with Dynasore, a specific inhibitor of dynamin, which is involved in endocytosis-mediated internalization of EV in many cell types [22]. The drug significantly reduced the entry of SyEV into macrophages, and incubation at 4 °C also blocked the uptake of SyEV (Fig. 4c). This suggests that SyEV are taken up actively and at least in part via endocytosis-mediated internalization and not by passive diffusion.

### Small peptides with a cholesterol anchor can be efficiently loaded into SyEV

**Fig. 5:**
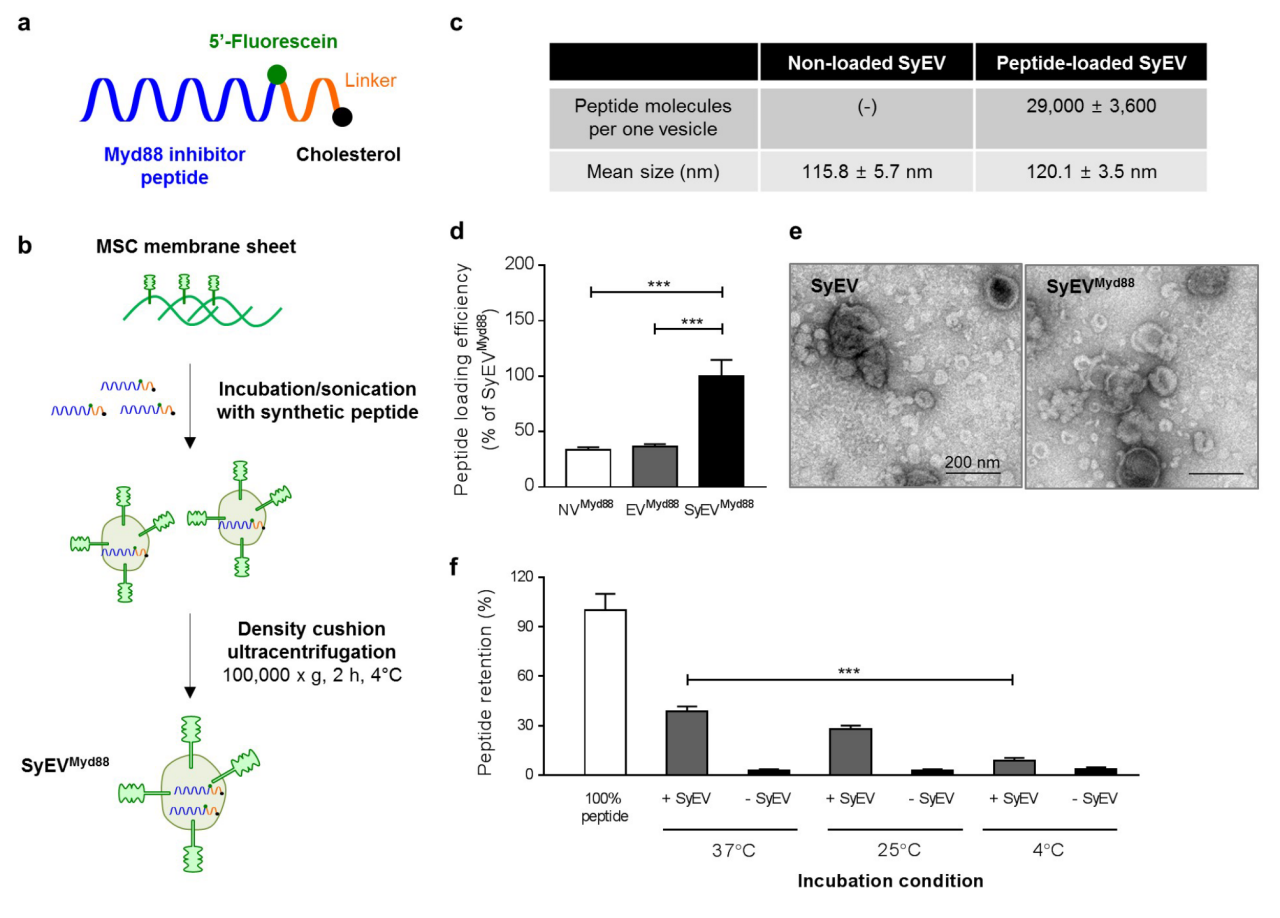
Generation and characterization of peptide-loaded SyEV. **a** Structure of fluorophore and cholesterol-conjugated peptides targeting Myd88 signaling molecules. **b** Flowchart of the SyEV loading procedure with the peptides. Co-incubation of MSC membranes and peptides via sonication resulted in the peptide-loaded SyEV (SyEV^Myd88^) that were then purified by iodixanol-based density gradient ultracentrifugation. **c** The characteristics of non-loaded and peptide-loaded SyEV, including the loading efficiency and vesicular size. **d** The loading efficiency of peptides in high pH-untreated NV/EV or treated SyEV (*n* = 3). **e** Morphology of SyEV^Myd88^ analyzed by TEM. Scale bars, 200 nm. **f** Optimization of conditions for loading of SyEV with peptides. The quantity of peptides in the final samples following ultracentrifugation was assessed by the fluorescent signal of samples containing SyEV (+ SyEV) or without SyEV (–SyEV) at various incubation temperatures (*n* = 3). Data are presented as the mean ± SEM. ^***^P < 0.001 by one-way ANOVA with Tukey’s post test

We used a commercially available Myd88-specific inhibitory peptide that has been previously shown to suppress the intracellular inflammatory pathway induced by the activation of various TLRs [23]. This peptide was additionally modified with a cholesterol anchor and fluorophore to associate the peptide to the SyEV membranes and to be able to quantify the number of peptides encapsulated within the SyEV (Fig. 5a). To generate peptide-loaded SyEV, high pH-treated MSC membranes were incubated with peptides under mild sonication and then subjected to iodixanol-based density gradient centrifugation to separate peptide-loaded SyEV (SyEV^Myd88^) from free peptides (Fig. 5b). Treatment with high pH enabled the efficient loading of peptides with SyEV membranes, resulting in approximately 29,000 peptides loaded per vesicle (Fig. 5c), whereas NV or EV not treated with high pH exhibited reduced loading efficiency (Fig. 5d). Nanoparticle tracking analysis and TEM were used to characterize the mean size and morphology of vesicles, and there were no observable differences between non-loaded SyEV and SyEV^Myd88^ (Fig. 5e), suggesting that the peptide loading process did not affect the integrity of the SyEV. Also, the treatment of SyEV^Myd88^ with proteinase K (PK) showed that 50% of total peptides are loaded in the luminal side of vesicles (Additional file 1: Fig. S9).

To establish the optimal incubation condition for the maximum loading of peptides, we next investigated the effect of different incubation temperatures on SyEV loading by co-incubation with peptides (Fig. 5f). This experiment was performed both in the presence and the absence of SyEV as a control for the possibility that increased temperature could result in the self-aggregation of peptides. Incubation at 4 °C resulted in low peptide binding to SyEV, implying that the SyEV membranes are less dynamic at low temperature. However, there was a significant 45% increase in the amount of peptides retained in the SyEV when the incubation temperature was 37 °C. In each control condition, very few peptides (2%) were obtained from the iodixanol gradient in the absence of SyEV.

### **SyEV**^**Myd88**^**exhibits greater protective activity in septic mice than free peptides and non-loaded SyEV**

**Fig. 6:**
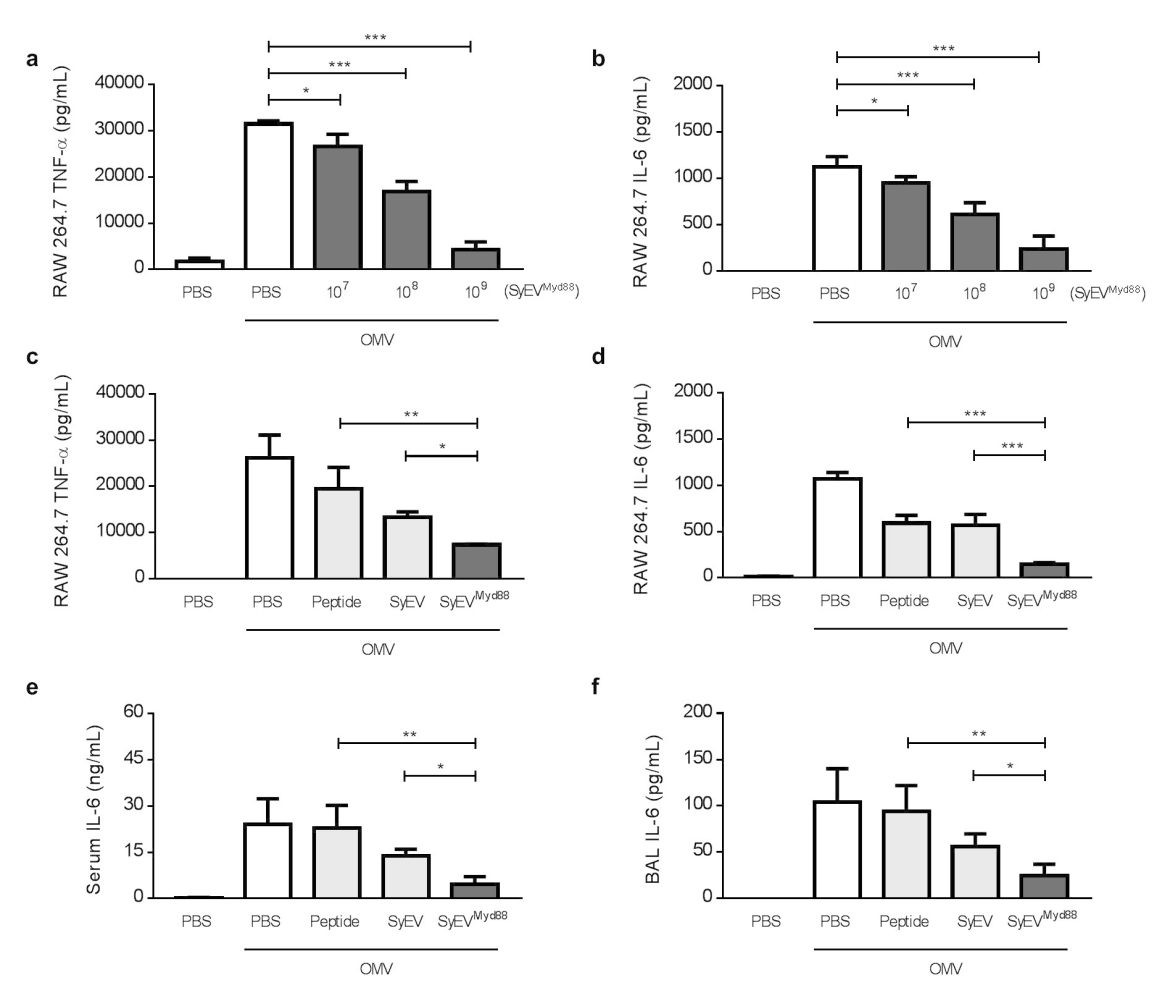
SyEV^Myd88^ shows greater therapeutic activity than single peptides or non-loaded SyEV. **a, b** RAW 264.7 cells were pre-treated with OMV (100 ng/mL) for 3 h and incubated with various doses of SyEV^Myd88^ for 15 h. The concentration of TNF-α (**a**) and IL-6 (**b**) in the conditioned media was measured (*n* = 3). **c, d** The anti-inflammatory activity of SyEV^Myd88^ was compared with that of non-loaded SyEV or peptides alone in RAW 264.7 cells. The macrophages were treated with peptides (1 µg), SyEV (1 × 10^9^), or SyEV^Myd88^ (1 × 10^9^) for 15 h, and then the concentration of TNF-α (**c**) and IL-6 (**d**) in the supernatants was quantified (*n* = 3). **e, f** In order to compare the therapeutic potential between groups in vivo, peptides (2 µg), SyEV (2 × 10^9^), or SyEV^Myd88^ (2 × 10^9^) were i.p. administered in OMV-induced septic mice (*n* = 5). The concentration of IL-6 in serum (**e**) and BAL fluid (**f**) was investigated at 6 h after OMV injection. Note that intact SyEV (1 × 10^9^) included 1 µg of peptides. Throughout, data are presented as the mean ± SEM. ^*^P < 0.05, ^**^P < 0.01, ^***^P < 0.001 by one-way ANOVA with Tukey’s post test

Our previous data showed that anti-inflammatory peptides can be efficiently associated with SyEV (Fig. 5), thus we asked whether the peptide-loaded SyEV can enhance the anti-inflammatory activity of peptides in the recipient cells. To first evaluate the dose response to SyEV^Myd88^ in the activated macrophages in vitro, RAW 264.7 cells were treated with different doses of SyEV^Myd88^ followed by measurement of cytokine production in the supernatants (Fig. 6a, b). The peptide-loaded SyEV dose-dependently inhibited OMV-induced cytokine release. To compare the therapeutic activity of SyEV^Myd88^ with free peptides and non-loaded SyEV, we next treated RAW 264.7 cells with single peptides alone or with the same particle number of non-loaded SyEV and SyEV^Myd88^ carrying an equivalent amount of peptide. SyEV^Myd88^ showed a significantly greater anti-inflammatory effect than both non-loaded SyEV and an equivalent amount of free peptides (Fig. 6c, d), illustrating the enhanced therapeutic potency of SyEV^Myd88^. Also, SyEV^Myd88^ were significantly more efficient to induce an anti-inflammatory effect compared to NV^Myd88^ or EV^Myd88^, indicating that SyEV are more optimal drug delivery vehicle to convey enhanced therapeutic potency (Additional file 1: Fig. S10).

We further exposed mice to OMV to assess the anti-inflammatory activity of SyEV^Myd88^ in vivo. The pro-inflammatory cytokine levels in both serum (Fig. 6e) and BAL fluid (Fig. 6f) showed greater anti-inflammatory efficacy with SyEV^Myd88^ than non-loaded SyEV. However, treatment with an equivalent amount of free peptides had no protective effects against OMV-induced cytokine release. These results suggest that SyEV^Myd88^ has a synergistic effect in inhibiting inflammation because the peptide treatment was inactive by itself.

## Discussion

In this study, we have shown that MSC-derived SyEV have intrinsic anti-inflammatory function and can also serve as a promising drug delivery platform to target severe inflammatory conditions. Specifically, the SyEV were produced via a multi-step process using extrusion and ionic stress to remove unwanted cytosolic components. The SyEV could be externally loaded with an anti-inflammatory peptide targeting intracellular Myd88, which is a key intracellular signaling molecule in innate immunity. Importantly, the peptide-loaded SyEV exhibited an additive reduction in OMV-mediated pro-inflammatory cytokine release from macrophages. In vivo, a highly efficacious and synergistic attenuation of OMV-derived cytokines in blood and BAL fluid was observed in mice treated with peptide-loaded SyEV, whereas the peptide alone had no effect, suggesting that the loaded SyEV are highly efficient in treating systemic inflammation.

In this study we created biologically therapeutic “EV-like” vesicles through a process of extrusion of whole MSC through a membrane to create NV [13] and exposure of these NV to high pH solution, which can open the membrane structure and discharge DNA, RNA, and cytosolic proteins from the inside the vesicles [14]. Synthetic drug delivery systems using liposomes, carbon nanotubes, or polymeric nanoparticles have been generally applied for the delivery of encapsulated therapeutics [24]. Despite promising efficacy results, these approaches face critical issues of low biocompatibility and scalability. However, SyEV can be generated in large quantities from parental cells and they are characterized by high biocompatibility, thus making them an optimal form of bio-inspired drug delivery. Also, we demonstrated that the SyEV production process is simple and rapid, which may be advantageous from the perspective of cost-effectiveness over traditional EV isolation. system. There are several reports to use extrusion or sonication for making nanoscale vesicles, which is a similar process to our technology. However, our SyEV platform has an advantage over previous methods to remove unwanted cytosolic components by opening and closing the vesicles, thus it is expected to increase the loading capacity for external drugs [25, 26]. Moreover, there is a risk that MSC could undergo malignant transformation if used as therapeutics due to gene instability, and this is an important limitation for MSC-based therapeutics [27, 28]. However, SyEV include little or no DNA and have no proliferation potential, suggesting that these types of vesicles are safer than cells as therapeutics, thus the SyEV platform is preferable for being utilized in a clinical setting.

Treatment with MSC-derived EV has been shown to mediate the repair of injured tissues and to modulate immune responses, and they are considered to be a promising cell-free therapy [29]. The beneficial effects and safety profile of EV have also been confirmed in patients with chronic kidney diseases [30] and severe inflammation [31]. We here confirmed that MSC-derived SyEV also exhibit high immuno-modulatory activity in vitro and in vivo, comparable to MSC-derived EV, even though the intravesicular protein components and DNA are significantly reduced in the manufacturing process. It is therefore likely that the therapeutic potential of SyEV can be ascribed to bioactive membrane proteins. Indeed, in this study MSC-derived SyEV were shown to retain the characteristic surface markers of EV (CD63, CD81 and Flotillin-1) and other membrane proteins, suggesting that SyEV share a key feature of protein composition with EV related to bioactive function. In fact, MSC-derived EV have been shown to elicit cardio-protection and increased survival in septic mice [32, 33], and it is therefore not surprising that SyEV can have a protective function in systemic inflammation because they share key membrane proteins with MSC-derived EV. In order to elucidate the molecular mechanism that underlies SyEV-mediated anti-inflammatory activity, it would be necessary to clarify which signaling pathways are involved in target cells and in mice by using certain inhibitors of the targeted signaling molecules. Moreover, GO-term analysis of SyEV revealed that a high portion of plasma membrane, endoplasmic reticulum, and lysosomal proteins contribute to SyEV composition. SyEV are like a mixture of various membranes, and the relative ratio of each different membrane vesicles should be further explored by using specific cell organelle markers.

Based on signaling pathway analysis of the SyEV proteome, we found that SyEV proteins are related to immunomodulation. It is, however, most likely that more than one protein is important for the intrinsic anti-inflammatory function of the SyEV. In addition to bioactive proteins, MSC-EV-associated nucleic acids have been shown to have a distinct beneficial effect in inflammatory disease models. For example, mRNA for angiopoietin-1 in EV has been associated with the immunomodulation of lung injury [34]. Interestingly, there is increasing evidence for the presence of miRNA in EV and for their highly therapeutic effects. We therefore cannot exclude the possibility of the involvement of mRNA/miRNA in SyEV-mediated protection, although most nucleic acids and relevant molecules are removed from SyEV during the vesicle manufacturing process.

EV can generally be taken up by the target immune cells, allowing the release of vesicular cargo molecules into the recipient cell’s cytoplasm [35]. The EV can adhere to the recipient cells via the binding of EV surface proteins to cellular receptors followed by endocytosis, phagocytosis, or micropinocytosis. Although the specific mechanisms of uptake of SyEV have not yet been elucidated like they have been for EV, we show here that the internalization of MSC-SyEV is partially blocked by Dynasore, an endocytosis-inhibiting molecule. This molecule specifically inhibits clathrin-mediated endocytosis [36], and the partial reduction of uptake suggests that this pathway is likely only one of multiple routes for SyEV uptake. This finding is in line with previous findings that MSC-derived extruded NV are also taken up into the recipient cell by dynamin-associated endocytosis [13]. Taken together, these results suggest that endocytosis is a major pathway of uptake of SyEV into immune cells, but there is still redundant uptake despite blocking of the endocytosis pathways. Thus, dissecting other uptake pathways for SyEV in general in target cells is important.

It is generally recognized that free peptides or proteins are highly susceptible to interstitial proteases and thus cannot be easily delivered to cells as free molecules. Also, such molecules are unable to effectively cross cellular membranes due to their charge, which is a critical limiting factor for their therapeutic applications. Recently, EV have been developed as drug delivery vectors in many animal disease models [37, 38]. Because efficient loading of biological cargo molecules into EV is a very crucial step, researchers have used different loading methods such as electroporation or overexpression of protein candidates in EV-producing cells[5]. However, these approaches have multiple shortcomings, including morphological changes in EV and a lack of effectiveness [39]. Furthermore, the yield of naturally released EV is another limiting factor in developing EV as delivery tools, and SyEV may have an advantage in this regard. To overcome this issue, we utilized the SyEV platform to efficiently load an anti-inflammatory MyD88-targeting peptide into SyEV without any apparent alterations in vesicular morphology. We suggest that the targeting of Myd88, the major signaling molecule for most TLRs, is rational because of its potential to efficiently attenuate downstream inflammatory pathways induced by multiple types of inflammatory or infectious agents. The anti-inflammatory function of the peptides has been well documented in several in vivo disease models. Liu et al. reported that this and similar peptides given in excessive doses suppress the activation of microglia and macrophages in the brain, thus providing significant neuroprotection [40]. Also, this peptide can protect against liver injury induced by neutrophil infiltration [41]. However, because these studies used very high doses of free peptide to obtain therapeutic efficacy in vivo, these peptides have been difficult to develop for clinical use. For peptide therapeutics to function as efficient drugs, efficient delivery systems to deliver the peptides intracellularly are required. In the present study, the peptide-loaded SyEV could provide potent protection against systemic inflammation when compared to approximately the same dose of free peptides or to non-loaded SyEV. This is likely explained by the SyEV efficiently delivering the peptide to the cytoplasmic compartments of appropriate recipient cells, including inflammatory cells such as macrophages and potentially structural cells such as endothelium and epithelium cells that can participate in the inflammatory cascade.

It will be important to carefully consider the exact route of administration of any EV therapeutics designed to target some disease. In this work, we targeted the abdominal cavity via i.p. injection because this is the origin of inflammation in this disease model. Based on our previous findings, the i.p. administration of vesicles led to their distribution throughout the whole body, specifically the lung, liver, and kidney [13]. Thus, in the same context, the fate of SyEV in disease models needs to be further clarified for therapeutic applications.

## Conclusion

Our study shows for the first time the anti-inflammatory activity of membrane-derived SyEV from MSC that are deficient in cytosolic proteins and DNA in a model of systemic inflammation. This suggests that the vesicular membrane proteins, even more than the cargo molecules, are crucial for the SyEV’ immunomodulatory activity. Our findings further demonstrate the efficient loading of peptide-based therapeutics in the process of manufacturing the SyEV and suggest that the SyEV and the anti-inflammatory peptide synergize in vivo to reduce inflammation to very low levels. MSC and MSC-EV are both being considered for the treatment of clinical inflammatory diseases, but their efficacy might be limited. We suggest here that MSC-derived SyEV can be employed as a highly efficient anti-inflammatory drug candidate when loaded with an appropriate anti-inflammatory molecule.

## Methods

### Animals

Wild-type mice of the C57BL/6 genetic background (6 weeks old) were obtained from Charles River. The mice were raised in the experimental animal room at the Experimental Biomedicine facility at the University of Gothenburg, Sweden. The experiment was approved by the local Animal Ethics Committee in Gothenburg, Sweden (permit no. Dnr 5.8.18–03598/2019) and was performed under institutional animal use and care guidelines.

### Cell cultures

Human bone marrow MSC were purchased from ATCC (Manassas, VA), and grown in Minimum Essential Media alpha GlutaMAX (Thermo Fisher Scientific, Waltham, MA) including 10% fetal bovine serum (FBS), 100 U/mL penicillin, and 100 µg/mL streptomycin. RAW 264.7 cells were maintained in Dulbecco’s modified Eagle’s medium (HyClone, Logan, UT) with 10% FBS, 100 U/mL penicillin, and 100 µg/mL streptomycin. All cells were cultured at 37 °C in an atmosphere of 5% CO_2_.

### Preparation of SyEV

MSC-derived SyEV were generated using the previous protocol with some modification [13]. The MSC (passage five) were resuspended at a density of 5 × 10^6^ cells per mL in 10 mL of phosphate-buffered saline (PBS). The suspension was sequentially passed five times through each of the membrane filters size (Whatman, Dassel, Germany) with a pore of 10 μm, 5 μm, and 1 μm, using a mini-extruder (Avanti Polar Lipids, Birmingham, AL). The total samples were applied to the top of iodixanol (Axis-Shield PoC AS, Oslo, Norway) gradient layers (1 mL of 50% and 2 mL of 10% iodixanol). The vesicles accumulated between the 50% and 10% iodixanol layers after ultracentrifugation at 100,000 × *g* for 2 h, and these were considered as NV. The NV were incubated with the same volume of high pH solution (200 mM Na_2_CO_3_, pH 11) for 1 h at 25 °C. The solution was loaded onto 4 mL of 50% iodixanol, followed by addition of 4 mL of 30% and 2 mL of 10% iodixanol in ultracentrifuge tubes. The layer formed between 10% and 30% iodixanol after ultracentrifugation at 100,000 × *g* for 2 h was collected. Finally, the vesicle samples were sonicated for 30 min with an ultrasonic bath of 44 kHz (Grant, Cambridge, UK), and these were considered SyEV.

### Isolation of EV

The MSC-derived supernatants were pelleted at 300 × *g* for 10 min and 2,000 × *g* for 20 min to remove cell debris. The resulting supernatants were sequentially ultracentrifuged at 16,500 × *g* for 20 min and 120,000 × *g* for 2.5 h to collect large (microvesicles) and small vesicles (exosomes), respectively. The fraction with the small exosomes was mixed with 4 mL of 50% iodixanol and then placed on an iodixanol gradient of 4 mL of 30% iodixanol and 2 mL of 10% iodixanol. The layer between 10% and 30% iodixanol after ultracentrifugation at 100,000 × *g* for 2 h was taken. The vesicles in this layer were considered EV.

### **Isolation of OMV derived from*Escherichia coli***

The bacterial cultures were centrifuged at 6,000 × *g* at 4˚C for 20 min, and then the supernatant was passed through a 0.45 μm vacuum filter followed by concentration in a Vivaflow 200 ultrafiltration module (Sartorius, Goettingen, Germany) using a 100 kDa cut-off membrane. The concentrated solution was subjected to ultracentrifugation at 150,000 × *g* at 4 °C for 3 h and resuspended in PBS.

### TEM

SyEV were visualized by negative staining for TEM. The vesicles were placed on glow-discharged 200-mesh formvar and carbon-coated copper grids (Electron Microscopy Sciences, Hatfield, PA) for 5 min. The samples were then rinsed with water followed by fixation using PBS supplemented with 2.5% glutaraldehyde and further staining with 2% uranyl acetate for 1.5 min. Negative-stained SyEV were analyzed by digitization on a LEO 912AB Omega electron microscope (Carl Zeiss SMT, Oberkochen, Germany) at 120 kV with a Veleta CCD camera (Olympus-SiS, Stuttgart, Germany).

### Nanoparticle tracking analysis

SyEV were diluted in PBS, and the number of vesicles was determined using ZetaView analyzer (Particle Metrix GmbH, Meerbuch, Germany). The analyses were performed in triplicate, and each data point was obtained from two stationary layers with five measurements in each layer. The sensitivity of the camera was adjusted to 70 in all measurements. Data were interpreted using ZetaView analysis software version 8.2.30.1.

### DNA and RNA analysis

DNA from NV and SyEV was separated using a Qiamp DNA Blood Mini kit (Qiagen, Hilden, Germany) according to the manufacturer’s protocol. RNA was isolated using a miRCUR RNA isolation kit for biofluids (Exiqon, Woburn, MA) according to the manufacturer’s protocol. One microliter of isolated DNA or RNA was analyzed for its quality, yield, and nucleotide length by capillary electrophoresis using an Agilent high-sensitivity DNA chip or Agilent RNA 6000 Nanochip on an Agilent 2100 Bioanalyzer® (Agilent Technologies, Palo Alto, CA).

### LC-MS/MS analysis

Aliquots containing 30 µg of each vesicle were digested with trypsin using the filter-aided sample preparation (FASP) method [42], and the digested peptides were collected by centrifugation followed by labeling with TMT 10-plex isobaric mass tagging reagents (Thermo Fisher Scientific, Waltham, MA) according to the manufacturer’s instructions. The combined labeled samples were pre-fractionated on the Dionex Ultimate 3000 UPLC system (Thermo Fischer Scientific, Waltham, MA) using a Waters XBridge BEH C18 column (3.0 mm × 150 mm, particle size 3.5 μm, Waters Corporation, Milford, MA), and all fractions were dried on a Speedvac followed by reconstitution in 3% acetonitrile and 0.2% formic acid for analysis. Each fraction was analyzed on an Orbitrap Fusion Tribrid mass spectrometer interfaced with an Easy-nLC 1200 (Thermo Fisher Scientific, Waltham, MA). Peptides were captured on the Acclaim Pepmap 100 C18 trap column (100 μm ⋅ 2 cm, particle size 5 μm; Thermo Fischer Scientific) and separated on an in-house packed C18 analytical column (75 μm ⋅ 30 cm, particle size 3 μm). Precursor ion mass spectra were analyzed at 120,000 resolution, and then the most intense precursor ions were fragmented at a collision energy setting of 35. The MS/MS spectra were recorded in ion trap mode with a maximum injection time of 40 ms and an isolation window of 0.7 Da. MS3 spectra for reporter ion quantitation were recorded at 50,000 resolution with HCD fragmentation at a collision energy of 60 using the synchronous precursor selection of the 7 most abundant MS/MS fragments, with a maximum injection time of 100 ms.

### Database search

Data analysis was performed with the Swissprot *Homo sapiens* database using Proteome Discoverer version 2.2 (Thermo Fisher Scientific, Waltham, MA). Mascot 2.5.1 (Matrix Science, London, UK) was used as the search tool with precursor mass tolerance of 10 ppm and fragment mass tolerance of 0.6 Da. One missed cleavage was accepted, mono-oxidation on methionine was set as a variable modification, and methylthiolation on cysteine was set as a fixed modification. Percolator was used for the validation of the identification results with a strict target false discovery rate of 1%, and proteins were only considered when they were identified in all replicates. The Panther classification system was used for protein class analysis and pathway classification (http://pantherdb.org/). Gene ontology (GO) analysis was performed using DAVID (https://david.ncifcrf.gov/) and the Funrich analysis tool. The mass spectrometry data were deposited to the ProteomeXchange Consortium via the PRIDE partner repository with the dataset identifier PXD033512.

### Flow Nano Analyzer

Different vesicle samples were incubated with PE Mouse Anti-Human CD63 antibody (BD Pharmingen, San Diego, CA) and analyzed using a Flow Nano Analyzer (NanoFCM Inc., Xiamen, China) according to the manufacturer’s protocol. Briefly, 50 µL of vesicles (10^10^ particles/mL) was mixed with 50 µL of antibody for 30 min at 37 °C and then washed with PBS by centrifugation at 100,000 × *g* for 20 min. The labeled vesicles were diluted within the optimal range of particle numbers and analyzed using the NanoFCM software (NanoFCM Profession V1.0).

### **Macrophage cytokines*in vitro***

RAW 264.7 cells were placed in a 24-well plate, and then OMV (100 ng/mL) were added for 3 h to induce pro-inflammatory cytokines. Various concentrations of EV, NV, and SyEV were used to treat the cells, and TNF-α, IL-6, IL-1β and IL-10 in the supernatants at 15 h were measured using a DuoSet ELISA Development kit (R&D Systems, Minneapolis, MN). For monitoring the NF-ĸB signaling pathway, THP1-Lucia cells were purchased from InvivoGen (Toulouse, France). The cells were derived from the THP-1 monocyte cell line transfected with luciferase driven by an NF-ĸB inducible promoter. The cells were cultured in RPMI 1640 medium supplemented with 10% FBS, 100 U/mL penicillin, 100 µg/mL streptomycin, 25 mM HEPES, and 100 µg/mL normocin/zeocin and seeded in a 96-well plate. OMV (100 ng/mL) were treated for 3 h followed by the addition of SyEV (1 × 10^9^) for 18 h.

### ***In vivo*experiment**

Mice were intraperitoneally administered with OMV (15 µg) to induce systemic inflammation following i.p. injection with NV or SyEV (2 × 10^9^), and they were administered again with same dose of NV or SyEV after 1 h. Mice were sacrificed after 6 h following anesthetization with i.p. injection of xylazine chloride (10 mg/kg; Bayer, Gothenburg, Sweden) and ketamine hydrochloride (100 mg/kg; Pfizer AB, Kent, UK). Body temperature was monitored by a thermometer (Bioseb, Chaville, France). Peritoneal lavage fluid, blood, and BAL fluid were obtained from the mice, and the supernatants were kept at − 80 °C for cytokine measurement following low-speed centrifugation. The pelleted cells were examined using light microscopy to count the total number of immune cells. The concentrations of pro-inflammatory cytokines in the blood were measured using a DuoSet ELISA Development kit.

### Uptake of SyEV by macrophages

SyEV were stained with DiO (Molecular Probes, Eugene, OR) for 30 min at 37 °C. RAW 264.7 cells labelled with Cellmask Deep Red (Thermo Fisher Scientific, Waltham, MA) were incubated with the fluorescent SyEV for 6 h. The cells were fixed with 4% formaldehyde and then permeabilized with 0.2% Triton X-100 followed by mounting with Prolong Gold antifade reagent (Thermo Fisher Scientific, Waltham, MA). The uptake profile was monitored by a confocal microscope (Zeiss Axio observer; Carl Zeiss, Oberkochen, Germany). For the uptake inhibitor treatment, RAW 264.7 cells were preincubated with Dynasore (Sigma Aldrich, St. Louis, MO) for 30 min and then treated with DiO-SyEV for 6 h. Flow cytometry was performed using a BD FACSVerse Flow Cytometer running BD FACSuit Software (BD Biosciences, San Jose, CA) and FlowJo Software (Tree Star Inc., Ashland, OR).

### Loading of SyEV with peptides

Inhibitory peptides targeting Myd88 were synthetized by JPT Peptide Technologies (Berlin, Germany). The peptide sequence was Arg-Asp-Val-Leu-Pro-Gly-Thr-Cys-Val-Asn-Ser-cholesterol, and fluorescein was attached to the Cys side chain. First, MSC-derived membranes, which originated from NV (3 × 10^11^) treated with a high pH solution as indicated above, were incubated with the fluorescent peptides (100 µg) at 37 °C for 1 h with sonication. Non-loaded peptides were separated from peptide-loaded SyEV by iodixanol-based ultracentrifugation (4 mL of 50%, 4 mL of 30%, and 2 mL of 10% iodixanol) at 100,000 × *g* for 2 h. Finally, the loaded vesicles between 10% and 30% iodixanol were collected (SyEV^Myd88^), and the peptide loading efficiency was calculated using a multimode microplate reader (Varioskan LUX, Thermo Fisher Scientific, Waltham, MA; 495 nm excitation and 520 nm emission wavelengths) based on a calibration curve for the peptide quantification. To investigate the distribution of the loaded peptides, SyEV^Myd88^ were treated with proteinase K (100 µg/mL) at 37 °C for 30 min, followed by separated from the free enzyme by ultracentrifugation. And then, the specific fluorescence for the peptides was measured to compare proteinase K-treated vs. non-treated SyEV^Myd88^.

### Statistical analysis

The results were expressed as the mean and standard error of the mean (SEM). Unpaired two-tailed Student’s *t*-test was performed to compare two groups. One-way ANOVA followed by Tukey’s multiple comparison test was used to evaluate the difference between multiple groups, and two-way ANOVA was applied to compare multiple groups with two independent variables followed by Tukey’s multiple comparison test. *P* < 0.05 was considered to be significant.

## Electronic supplementary material

Below is the link to the electronic supplementary material.

Supplementary Material 1

Supplementary Material 2

## Data Availability

The data that support the findings of this study are available from the corresponding authors upon reasonable request.

## Abbreviations

MSC: Mesenchymal stem cells
EV: Extracellular vesicles
NV: Nanovesicles
SyEV: Synthetic eukaryotic vesicles
TLR: Toll-like receptors
OMV: Outer membrane vesicles
TEM: Transmission electron microscopy
TNF: Tumor necrosis factor
IL: Interleukin
NF-ĸB: nuclear factor-ĸB
i.p.: Intraperitoneally
BAL: Bronchoalveolar lavage
PK: Proteinase K
FBS: Fetal bovine serum
PBS: Phosphate-buffered saline
