# Supplementary figures and images for: Targeting Myd88 using peptide-loaded mesenchymal stem cell membrane-derived synthetic vesicles to treat systemic inflammation
Source: J Nanobiotechnology. 2022 Oct 15;20:451. doi: 10.1186/s12951-022-01660-x (PMC9571445; doi:10.1186/s12951-022-01660-x)

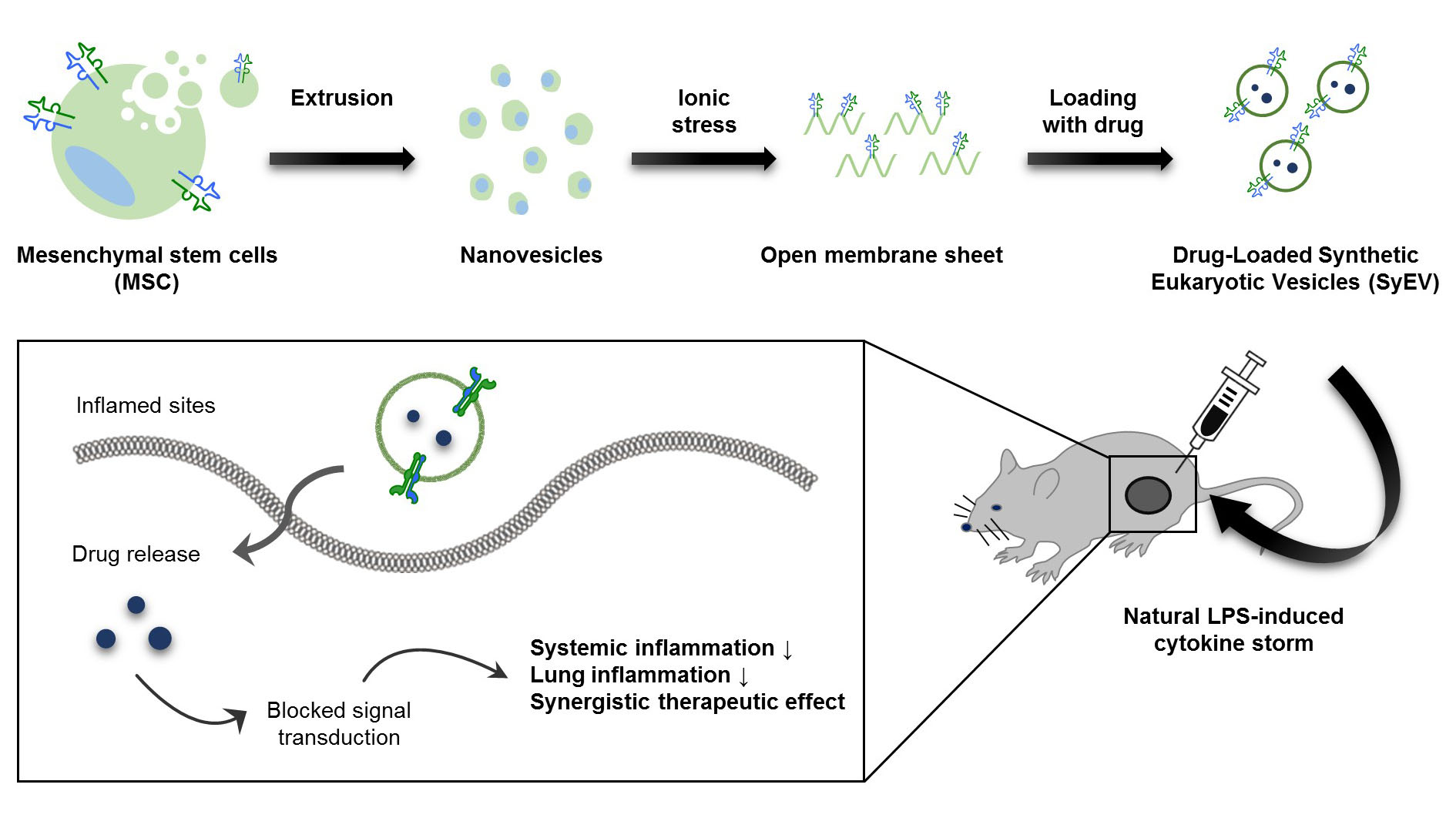

Supplement: Supplementary file 1 — Supplementary Material 1 [file 12951_2022_1660_MOESM1_ESM.jpg]
