# Supplementary material for: Targeting Myd88 using peptide-loaded mesenchymal stem cell membrane-derived synthetic vesicles to treat systemic inflammation
Source: J Nanobiotechnology. 2022 Oct 15;20:451. doi: 10.1186/s12951-022-01660-x (PMC9571445; doi:10.1186/s12951-022-01660-x)
Supplement: Supplementary file 2 — Supplementary Material 2 [file 12951_2022_1660_MOESM2_ESM.docx]

**
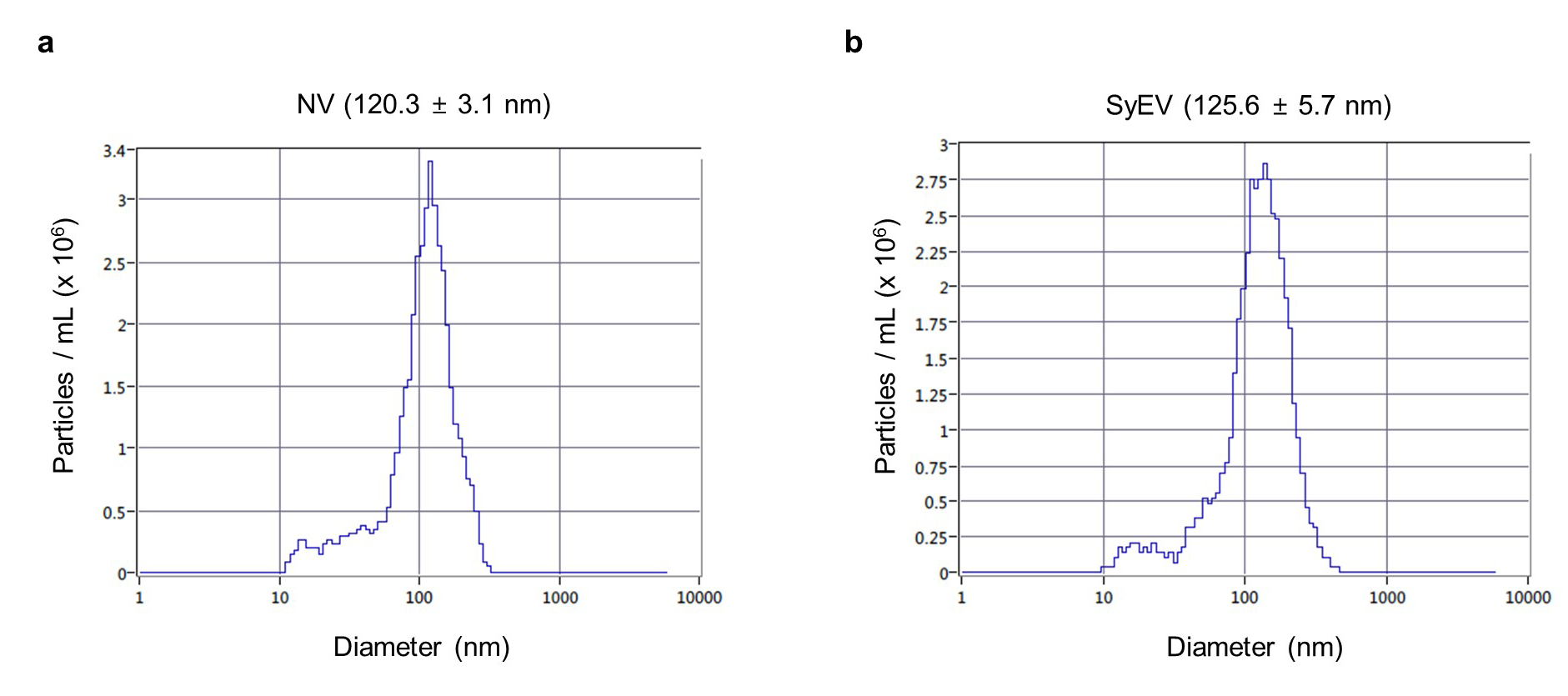
**

**Figure S1** Characterization of SyEV. The size distribution of NV (**a**) and SyEV (**b**) measured by nanoparticle tracking analysis.


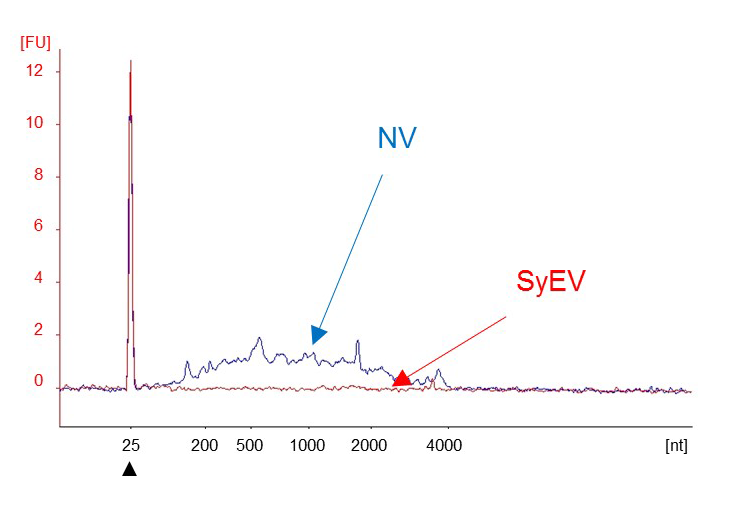


**Figure S2** Representative electropherograms of RNA molecules derived from SyEV in comparison to those from NV. Filled triangle indicates internal marker.

**
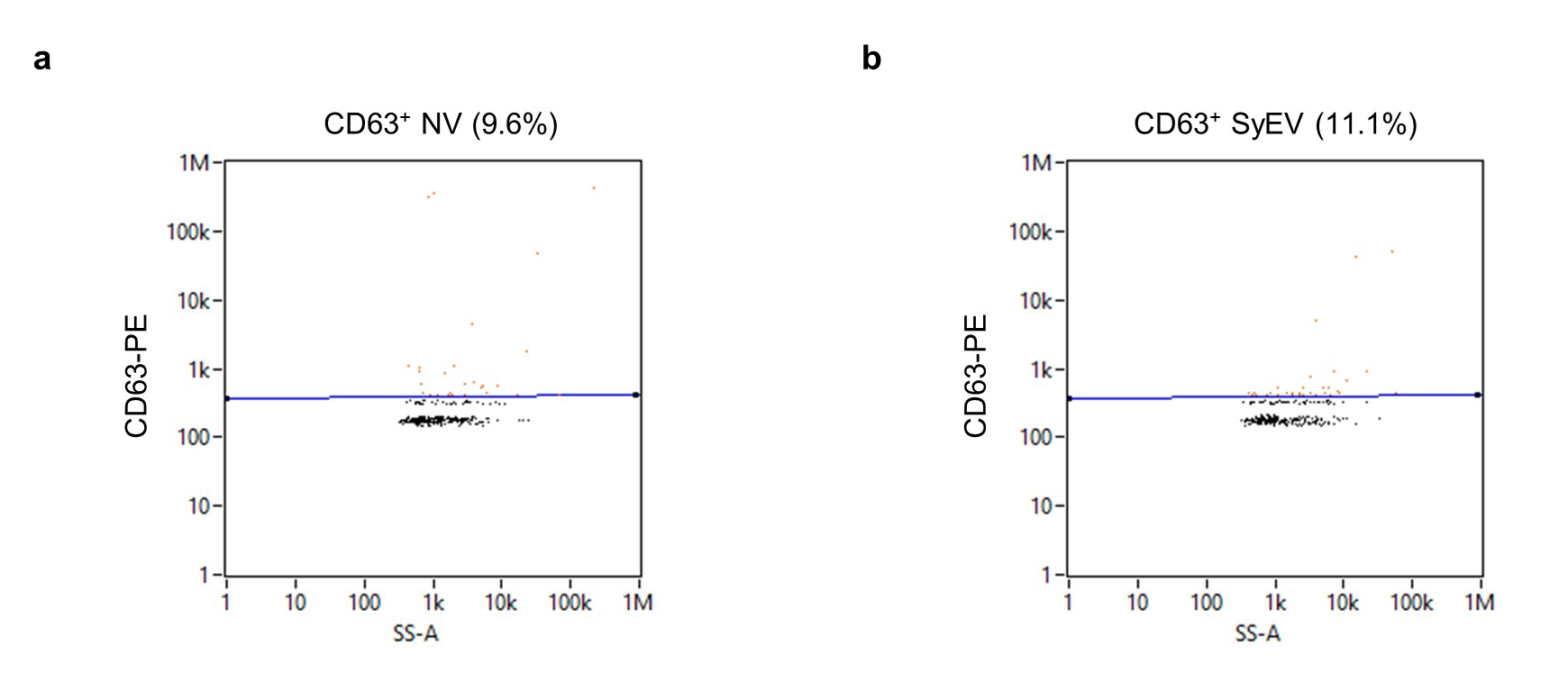
**

**Figure S3** The confirmed vesicular marker protein on SyEV. Expression of the vesicular surface protein CD63 on NV (**a**) and SyEV (**b**) identified by NanoFCM analysis.

**
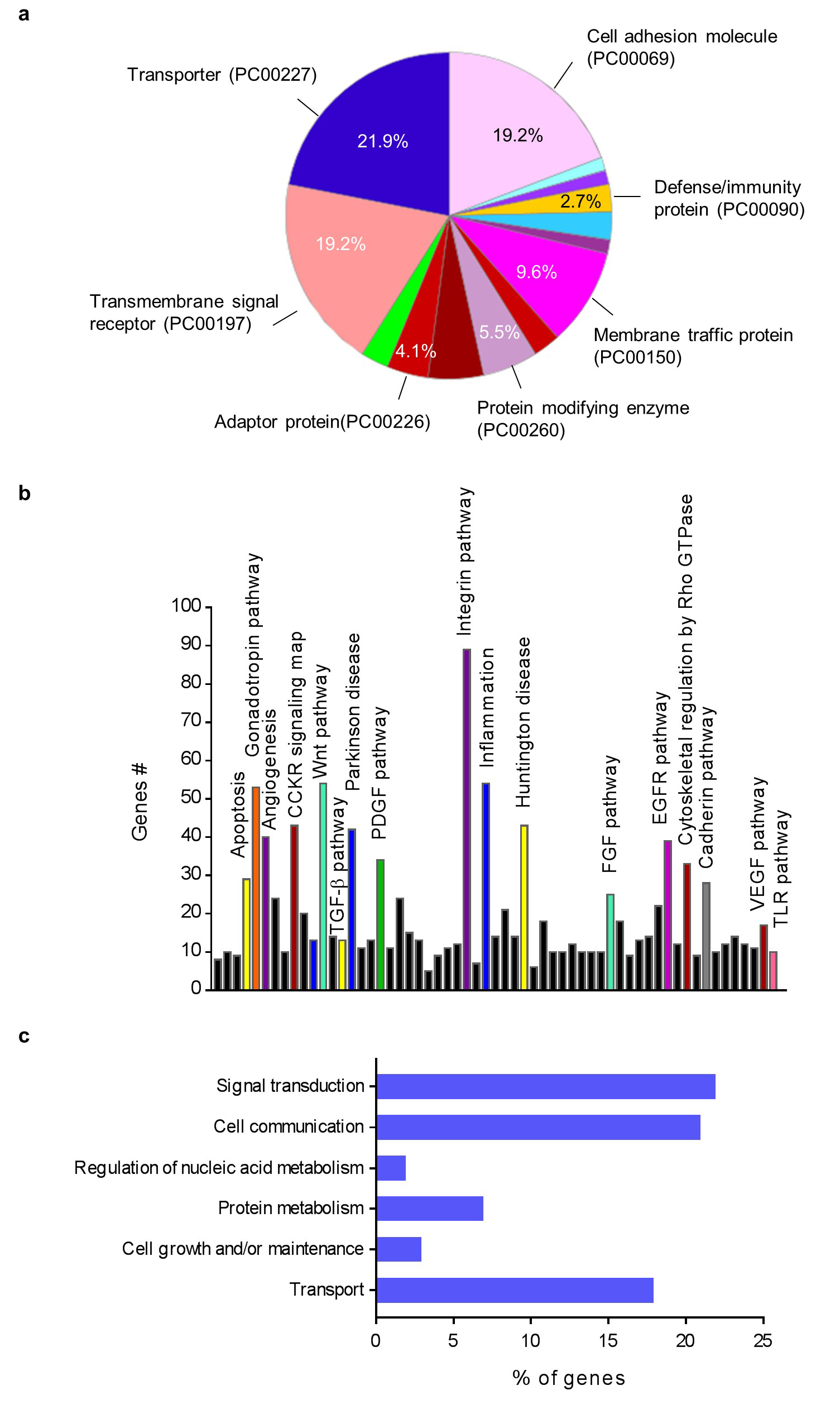
**

**Figure S4** Functional analysis of the MSC-SyEV proteome shows therapeutic potential on the immune system. **a** The distribution of the 3484 proteins identified from all of the SyEV proteins in different protein classes by Panther analysis. **b** Panther pathway analysis of the SyEV proteome. The representative pathways regarding EV function are indicated in the figure. **c** GO analysis of biological functions of SyEV proteins using the Funrich software. The bar chart indicates the relative percentage of genes in each functional category (*P* < 0.05).

**
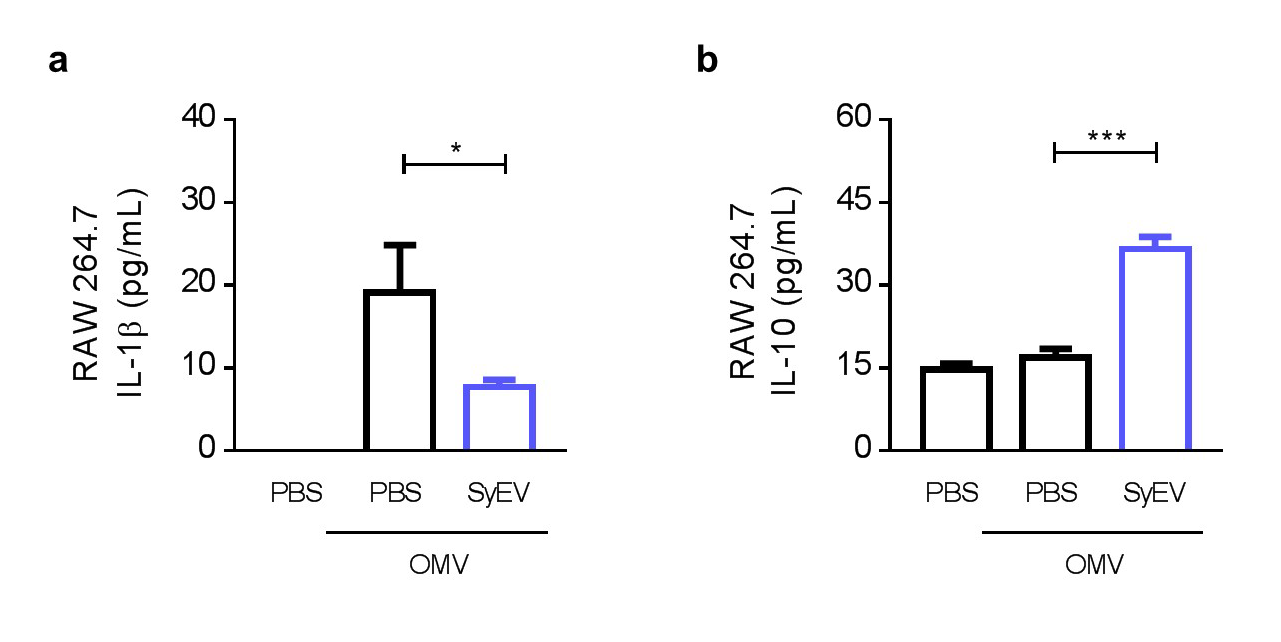
**

**Figure S5** The effect of SyEV on OMV-activated RAW 264.7 cells. RAW 264.7 cells were pre-treated with OMV (100 ng/mL) for 3 h and then incubated with SyEV for 15 h. The concentration of IL-1β (**a**) and IL-10 (**b**) was measured in the conditioned media (*n* = 3). Data are presented as the mean ± SEM. ^*^*P* < 0.05, ^***^*P* < 0.001 by one-way ANOVA with Tukey’s post test.


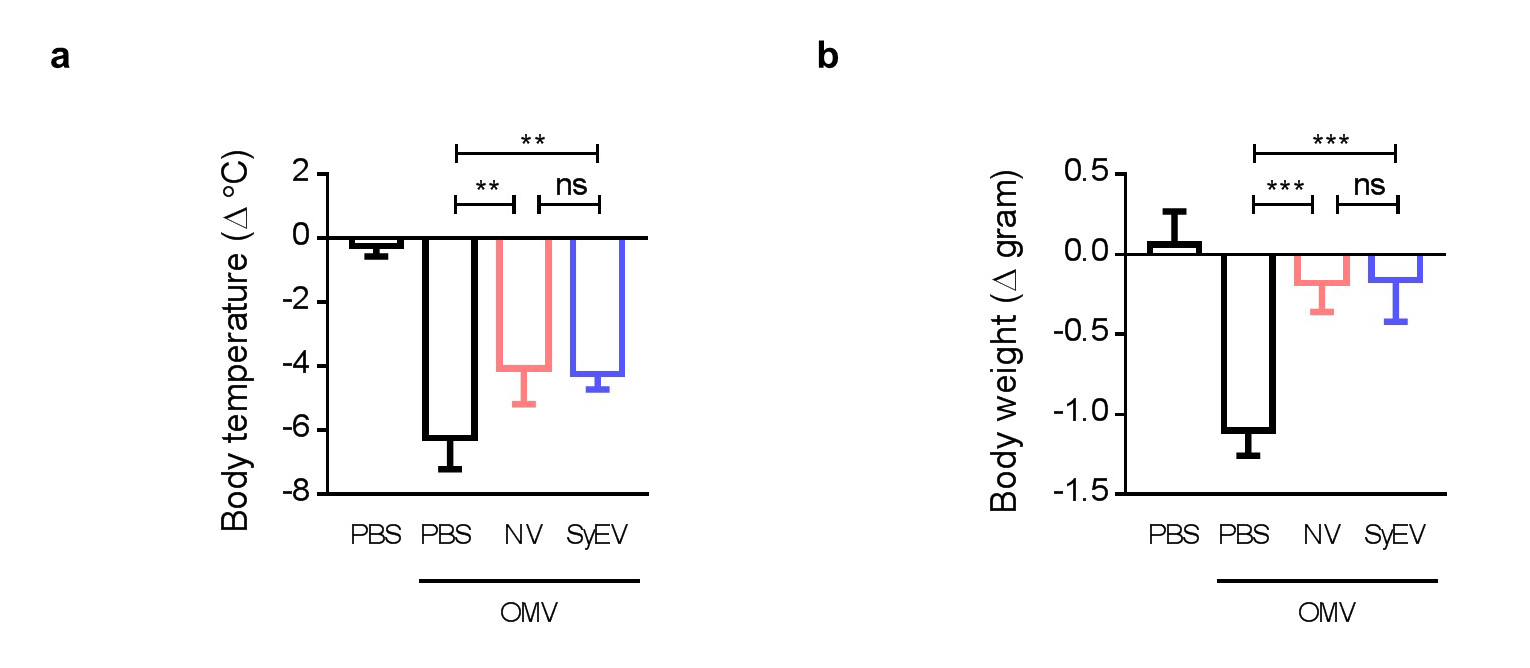


**Figure S6** The therapeutic activity of SyEV in septic mice is comparable to NV. Body temperature (**a**) and weight (**b**) were measured at 6 h following OMV administration in mice (*n* = 5). Data are presented as the mean ± SEM. ^**^*P* < 0.01, ^***^*P* < 0.001; ns, not significant, by one-way ANOVA with Tukey’s post test.


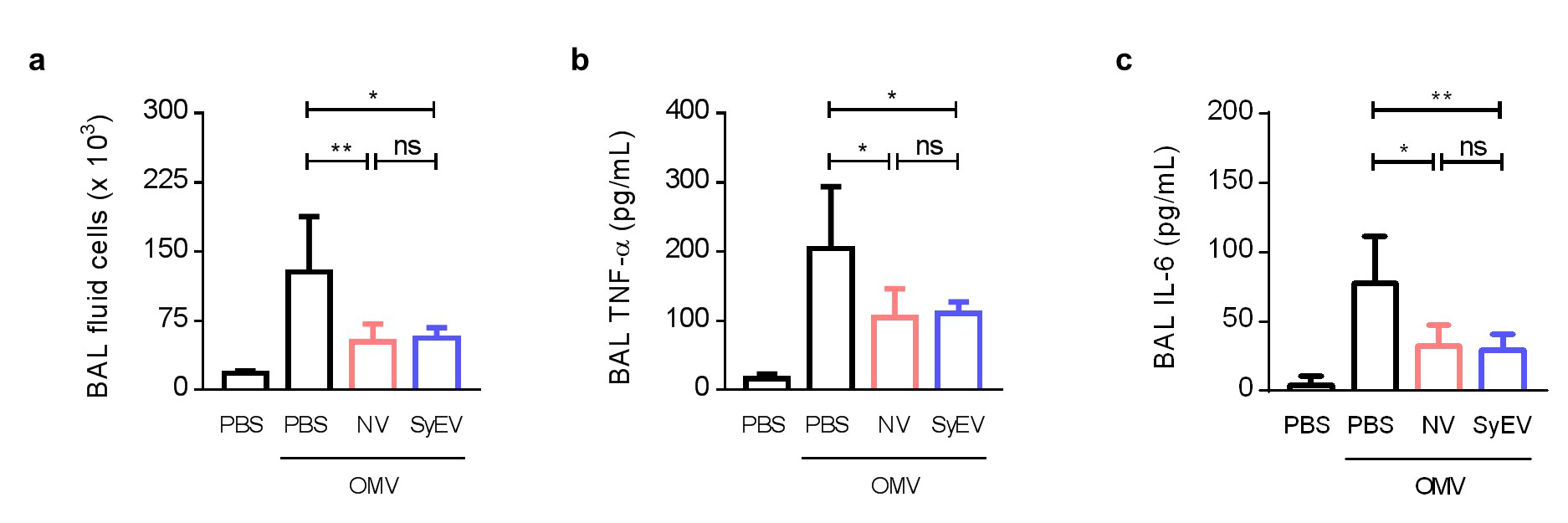


**Figure S7** Inflammatory cell count and cytokine level in BAL fluid of septic mice injected with SyEV or NV. **a** The total number of immune cells was counted in BAL fluid at 6 h following OMV injection (*n* = 5). **b, c** The effects of NV and SyEV on the concentrations of TNF-α (**b**) and IL-6 (**c**) were analyzed in BAL fluid (*n* = 5). Data are presented as the mean ± SEM. ^*^*P* < 0.05, ^**^*P* < 0.01; ns, not significant, by one-way ANOVA with Tukey’s post test.


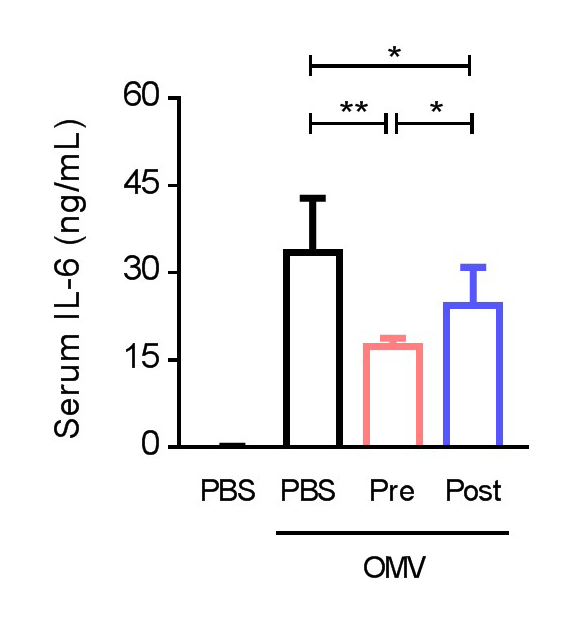


**Figure S8** Mice were i.p. injected with SyEV (2 × 10^9^) at 1 h before or after administration of OMV. Six hours after OMV injection, mice were sacrificed to measure the concentration of IL-6 in the serum (*n* = 5). Data are presented as the mean ± SEM. ^*^*P* < 0.05, ^**^*P* < 0.01 by one-way ANOVA with Tukey’s post test.


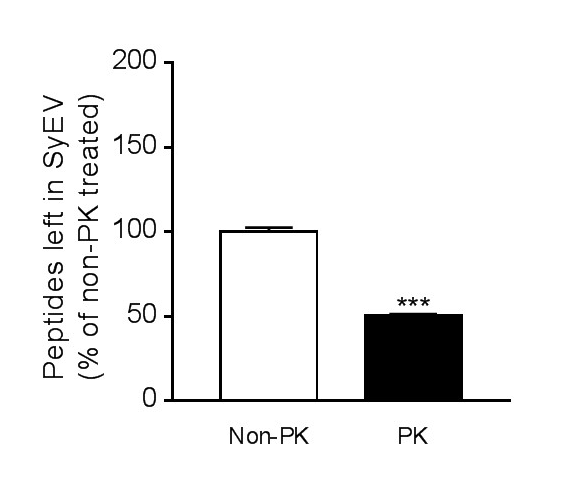


**Figure S9** The distribution of loaded peptides in SyEV. SyEV^Myd88^ were treated with proteinase K (PK) to digest peptides bound outside of the vesicles, and then the remaining peptide concentration was quantified using fluorometric approach (*n* = 3). Data are presented as the mean ± SEM. ****P* < 0.001 by unpaired two-tailed Student’s *t*-test.


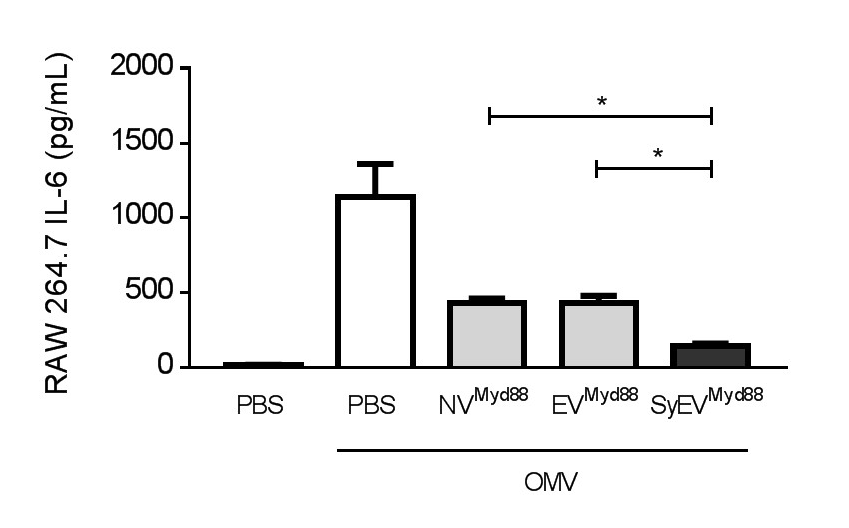


**Figure S10** The anti-inflammatory activity of SyEV^Myd88^ was compared with that of NV^Myd88^ or EV^Myd88^ in RAW 264.7 cells. The macrophages were treated with same particle numbers (1 × 10^9^) for 15 h, and then the concentration of IL-6 in the supernatants was quantified (*n* = 3). Data are presented as the mean ± SEM. ^*^*P* < 0.05 by one-way ANOVA with Tukey’s post test.
